# Supplementary material for: Onset of strong Iceland-Scotland overflow water 3.6 million years ago
Source: Nat Commun. 2025 May 9;16:4323. doi: 10.1038/s41467-025-59265-5 (PMC12064707; doi:10.1038/s41467-025-59265-5)
Supplement: Supplementary file 1 — Supplementary Information [file 41467_2025_59265_MOESM1_ESM.pdf]

# SUPPLEMENTARY INFORMATION

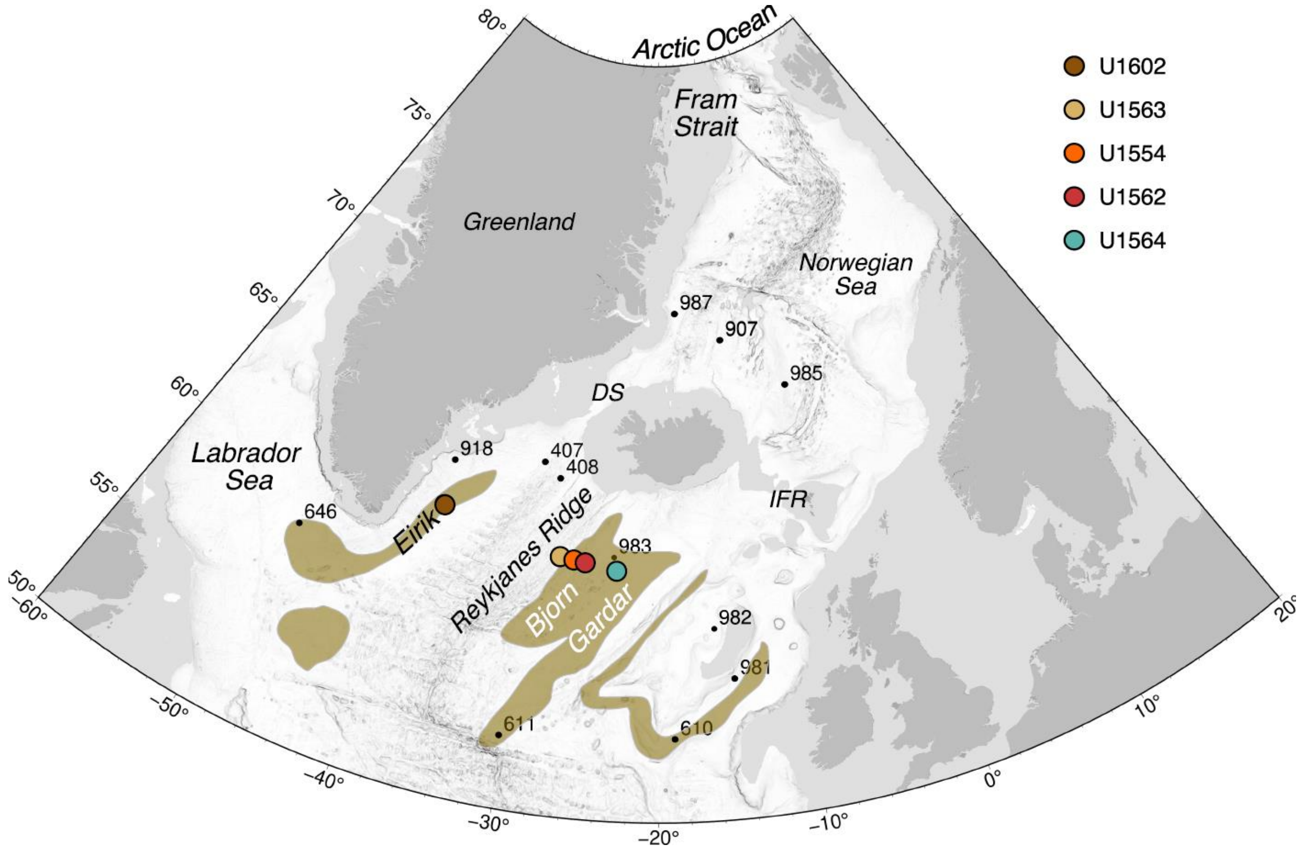


**Figure S1: Geographic locations of North Atlantic sites discussed in Table S1, as well as other sites discussed in the Supplementary Information.**

The main manuscript illustrates changes in lithology and sedimentation rates for IODP Expedition 395C and 395 Sites U1602, U1563, U1554, U1562, U1564 and ODP Site 982 on a ~60°N West–East transect. Here, we briefly synthesize DSDP/ODP/IODP sites in the North Atlantic Ocean that have: (i) a relevant geographic location (**Fig. S1**), (ii) continuous or semi-continuous core recovery across the Pliocene–Pleistocene, (iii) changes in sedimentation rate and/or lithology close to the 3.6 Ma transition and (iv) a robust age model. The idea of this overview is to document what records are available for comparison, not to claim that each of these records necessarily shows the same changes as documented by Expeditions 395C and 395. Overall the observed changes in these sites are consistent with the observations and interpretations presented in the main manuscript. The main observations regarding changes in sedimentation rates and lithology described below are sourced by the initial site reports (**Table S1**), with additional information cited in the text.

**Table S1: Summary of investigated older DSDP, ODP and IODP (U) sites**

| Number | Site/Leg-Exp | Location | Depth (m) | Drift sedimentation | Pliocene changes in sedimentation rates | Initial report |
|---|---|---|---|---|---|---|
| **1** | DSDP 407/49 | West of Iceland | ~2470 | Non-drift | Erosional hiatus across the Pliocene/Pleistocene | Luyendyk et al., 1979 (*1*) |
| **2** | DSDP 408/49 | West of Iceland | ~1620 | Non-drift | Erosional hiatus or condensation across the Pliocene/Pleistocene | Luyendyk et al., 1979 (*1*) |
| **3** | ODP 610/94 | Western Rockall Trough | ~2420 | Feni Drift | No significant change | Ruddiman et al., 1987 (*4*) |
| **4** | ODP 611/94 | South Gardar Ridge | ~3200 | South tip of Gardar Drift | No significant change | Ruddiman et al., 1987 (*4*) |
| **5** | ODP 645/105 | Baffin Bay | ~2000 | Non-drift | Core recovery gaps in the Pliocene | Srivastava et al., 1987 (*6*) |
| **6** | ODP 646/105 | South of Greenland | ~3450 | Tip of Eirik Drift | Abrupt increase in siliceous microfossils ~3.6 Ma | Srivastava et al., 1987 (*6*) |
| **7** | ODP 907/151 | Norwegian/Greenland Sea | ~1800 | Non-drift | Doubling in sed. rates ~3.6 Ma | Myhre et al., 1995 (*9*); Jansen et al., 1996 (*12*) |
| **8** | ODP 918/152 | Southeast Greenland margin | ~1880 | Non-drift | Tripling in sed. rates ~3.6 Ma | Larsen et al., 1994 (*10*) |
| **9** | ODP 981/162 | Southeast of Rockall bank | ~2180 | Feni Drift | No significant changes | Jansen et al., 1996 (*12*) |
| **10** | ODP 985/162 | Norwegian/Greenland Sea | ~2800 | Non-drift | Doubling in sed. rates ~3.6 Ma | Jansen et al., 1996 (*12*) |
| **11** | ODP 987/162 | Norwegian/Greenland Sea | ~1680 | Non-drift | No significant changes | Jansen et al., 1996 (*12*) |
| **12** | U1308/303 | Eastern North Atlantic | ~3520 | Non-drift | Doubling in sed. rates ~3.6 Ma | Channell et al., 2006 (*13*) |

**1, 2: DSDP Sites 407 and 408** are located west of Iceland. Both are characterized by hiatuses and a lithological transition from Pliocene nannofossil chalk or ooze to Pleistocene calcareous sandy muds. The latter has been interpreted as a consequence of intensified northward flowing bottom currents (*1*).

**3: ODP Site 610** is located at the Feni Drift and has been established as a drift sediment body deposited by the Norwegian Sea Overflow (*2*,*3*). Constant sedimentation rates are observed for the Quaternary and the Pliocene, and no significant changes occur ~3.6 Ma, while lithologies transition from nannofossil oozes and chalks to interlayered calcareous muds and nannofossil oozes during the late Pliocene (*4*). An interval with elevated biogenic silica (>10%) has been recorded close to the Early to Late Pliocene transition (*4*), in agreement with our observations. The relatively low sedimentation rates (~50 m/Myr) and the carbonate-rich lithologies suggest that sedimentation at this site was probably not controlled by drift deposition.

**4. ODP Site 611** is located in the southeastern tip of Gardar Drift and has been suggested to record drift sedimentation and deep overflow water activity from the Norwegian Sea (*5*). Sedimentation rates do not demonstrate any significant change within the Pliocene but are significantly reduced during the latest Pleistocene, while lithologies transition from nannofossil oozes and chalks to interbedded muds, calcareous muds and nannofossil oozes during the Late Pliocene (*4*). An interval with common biogenic silica is observed during the mid to Late Pliocene, concomitant with our observations at Gardar Drift Site U1564 (*4*; **Fig. 2**).

**5: ODP Site 645** is located in Baffin Bay and spans the age interval of interest but has a poorly resolved age model and a relatively large core recovery gap around 3.6 Ma between lithological units III and II (*6*).

A recent palynological study by *(7)* interestingly suggests the age of the top of lithological unit III to be close to 3.6 Ma, with the palynology indicating a rather warm and nearshore palaeoenvironment, and markedly colder palaeoenvironment with stratified surface waters for the overlying unit II. The results acquired by IODP Expedition 400 will likely better constrain Plio-Pleistocene palaeoenvironmental changes in the Baffin Bay area.

**6: ODP Site 646** is located towards the southern tip of the Eirik Drift and features similar observations as at Site U1602: (i) a sudden appearance of siliceous microfossils at ~3.6 Ma (or ~340 mbsf) and a sustained high rate of sedimentation indicating the drift at this location was already depositing at a high rate at this time (*6*). Downhole logging data suggests a corresponding lithological change at ~340 mbsf (*8*). We note that the palaeomagnetic reversal data are based on relatively low applied fields and show significant time gaps.

**7: ODP Site 907** is located in the SW part of the Norwegian–Greenland Sea, the source area of ISOW, and shows at ~3.6 Ma a doubling in sedimentation rate from about 10 m/Myr to 20 m/Myr, accompanied with a clear lithological change characterized by the disappearance of biogenic sediments and a marked increase in the > 2 mm siliciclastic sediment fraction (i.e., transition between lithological units III and II in *(9)*).

**8: ODP Site 918** is located near the southeast Greenland margin and shows a tripling of sedimentation rate from about 50 m/Myr to 150 m/Myr around 3.6 Ma (*10*). Core recovery in this interval is very low, making a detailed lithological investigation challenging, but a marked increase in coarse sand ice-rafted debris (IRD) has been documented from ~3.6 Ma, with smaller peaks in IRD starting earlier (*11*).

**9: ODP Site 981** is located on the Feni Drift and shows an increase in sedimentation rates (~50%) during the early Pliocene (~3.7 Ma), but no significant change in lithology, which is predominantly comprised of nannofossil ooze with varying amounts of clay (*12*).

**10: ODP Site 985** is located in the southern part of the Norwegian–Greenland Sea, also a source area of ISOW, and shows a doubling in sedimentation rate from 10–15 m/Myr to 20–25 m/Myr at ~3.6 Ma, accompanied by a subtle lithological transition characterized by an increase in biogenic carbonate content as well as the appearance of dropstones (i.e., transition between lithological units III and II in *12*).

**11: ODP Site 987** is located just north of the Denmark Strait in the Norwegian–Greenland Sea and does not show a pronounced change in lithology or sedimentation rate at ~3.6 Ma (*12*). Microfossil preservation is poor and therefore cannot be used as an age-control for the older part of the palaeomagnetic interpretation (Early Pliocene), limiting the robustness of sedimentation rate estimates.

**12: IODP Site U1308 (DSDP Site 609 (Leg 94))** is located in the eastern North Atlantic and shows a doubling in sedimentation rates at ~3.6 Ma and an abrupt increase in siliceous microfossils, but no other pronounced lithological changes (*13*).

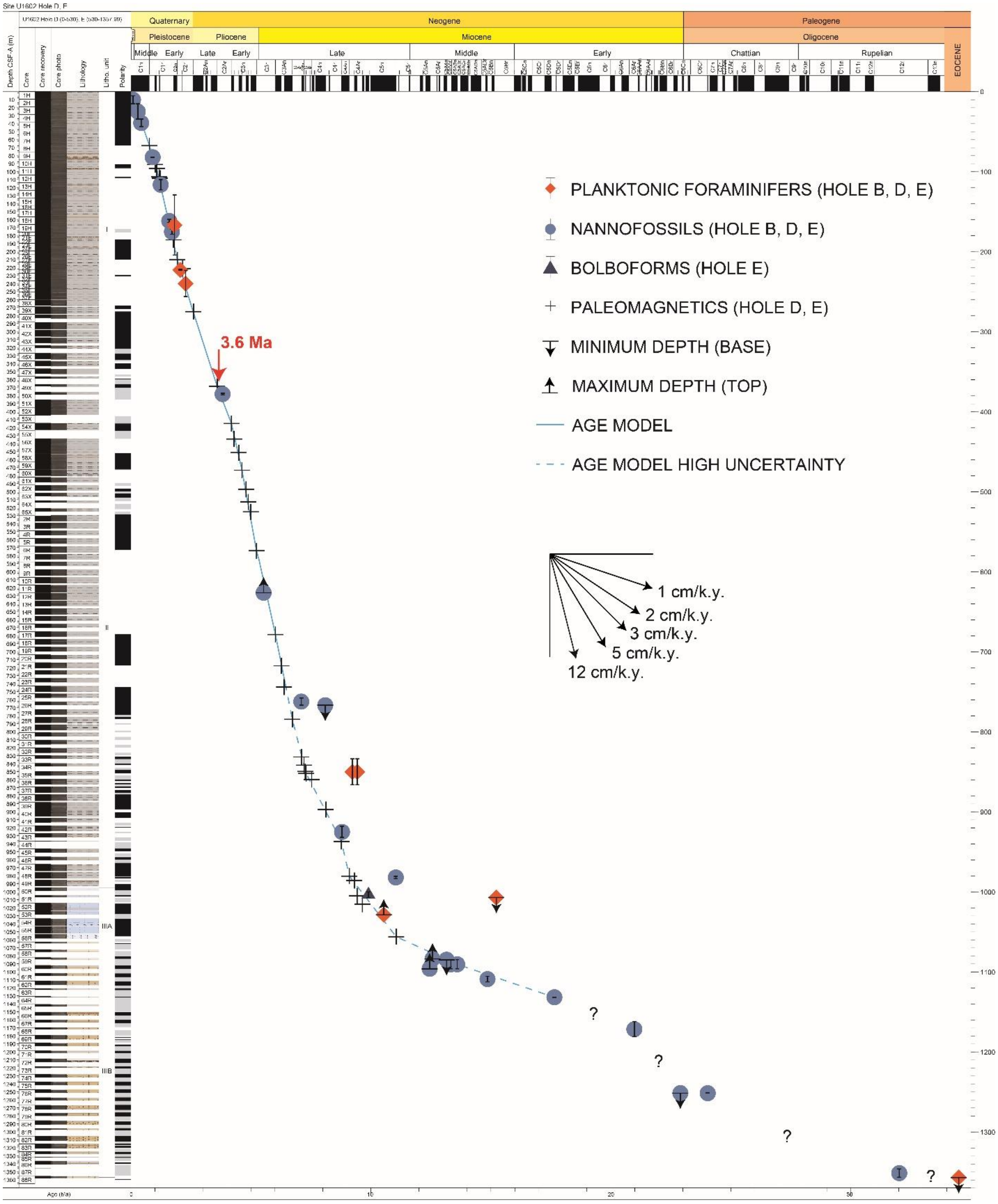


**Figure S2**: Ship-board age model for IODP Site U1602 based on biostratigraphy and palaeomagnetism. The arrow at 3.6 Ma is added for reference. The site shows relatively uninterrupted continuous contourite deposition although a small decrease in sedimentation rate and subtle changes in sedimentary components such as siliceous plankton do occur around 3.6 Ma (discussed further in *ref. 18*).

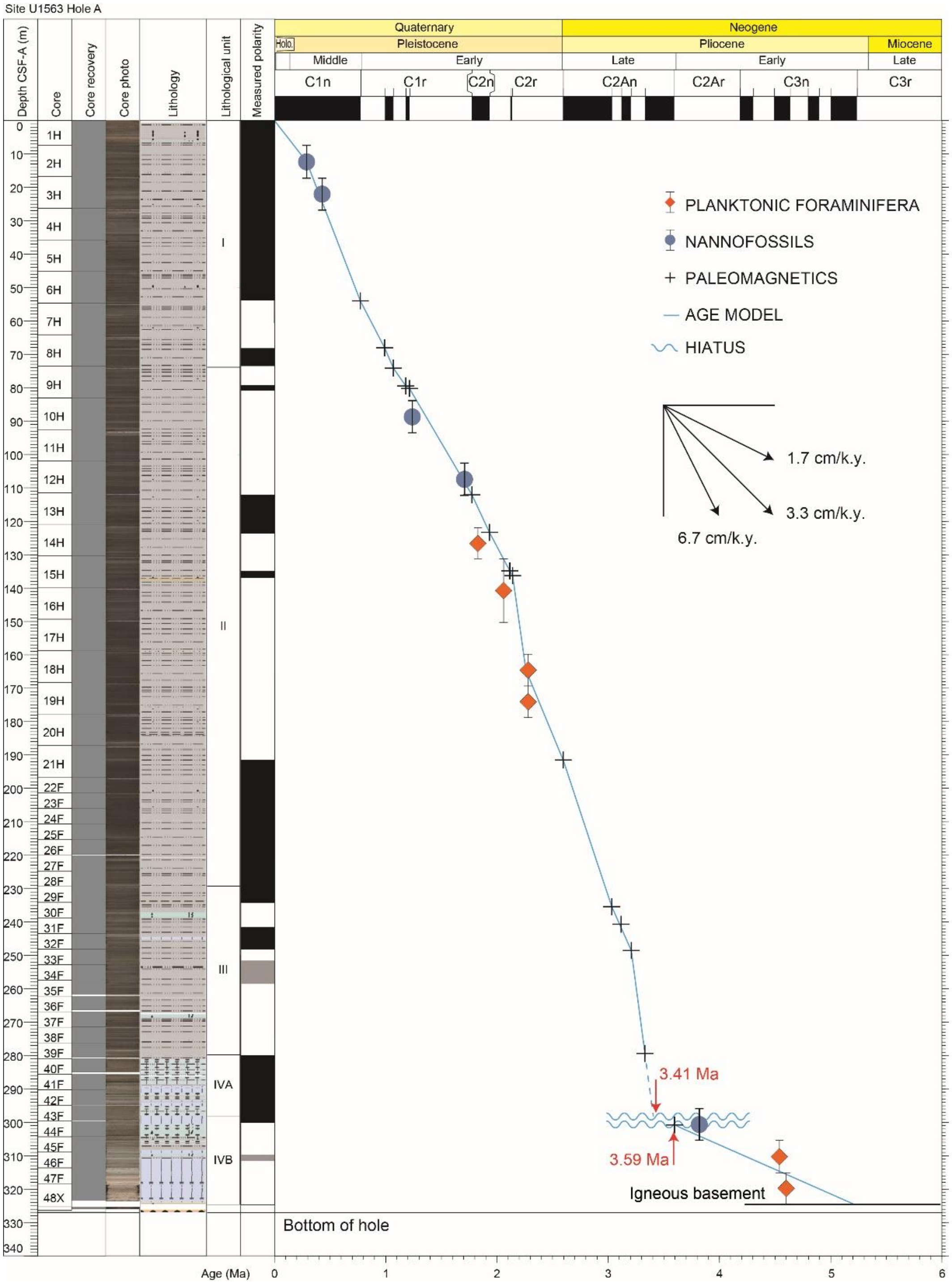


**Figure S3**: Shipboard age model for IODP Site U1563 based on biostratigraphy and palaeomagnetism. The arrows at 3.41 Ma and 3.59 Ma are added for reference. This shows the level of sedimentary complexity highlighted between two inferred hiatus lines, with the projected ages of sediment above (3.4 Ma) and below (3.6 Ma) this zone highlighted with red arrows. The site shows a sharp change in sedimentation rate associated with a lithological change from calcareous to clastic dominated sedimentation (discussed further in *ref. 19*).

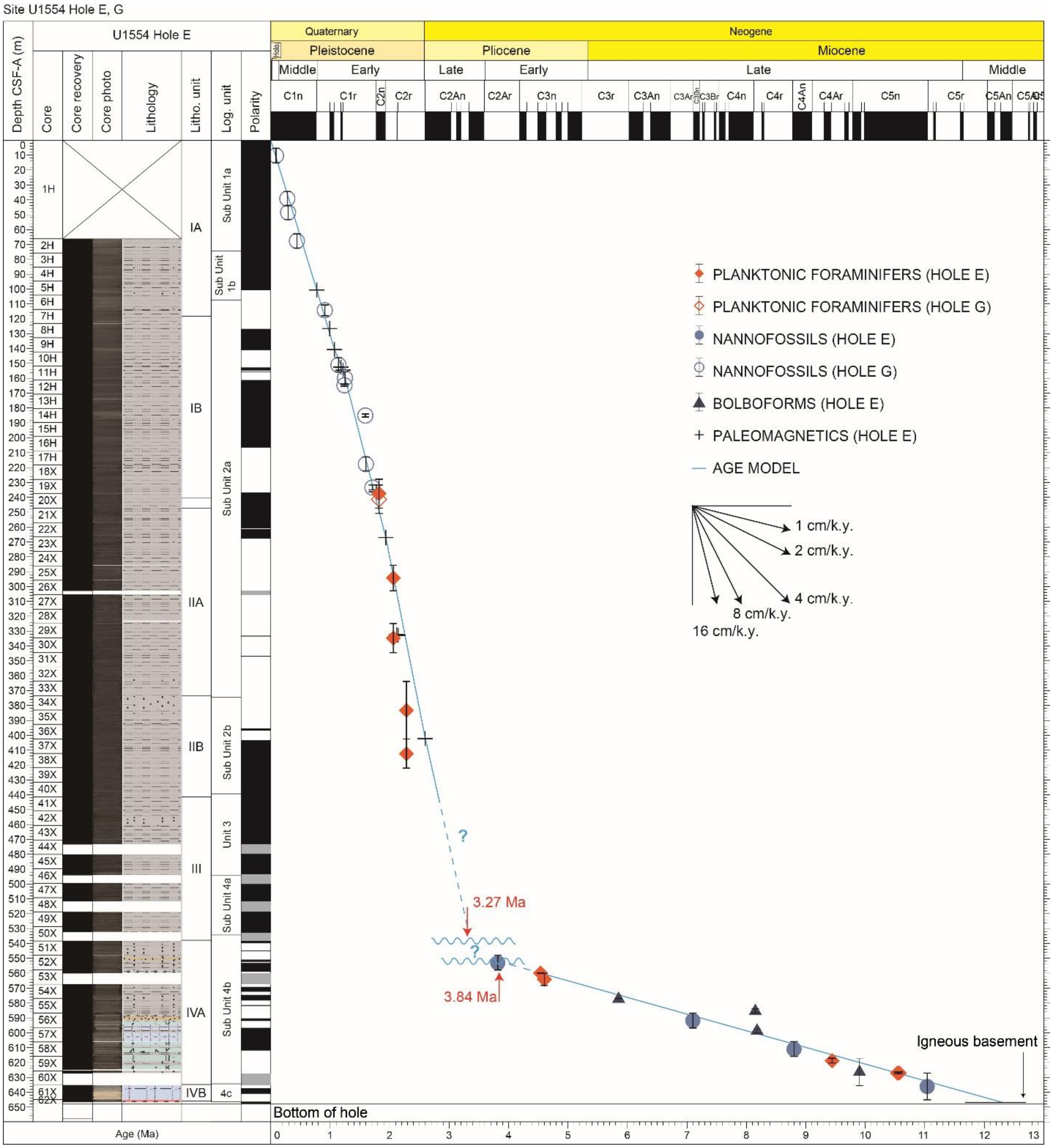


**Figure S4**: Shipboard age model for IODP Site U1554 based on biostratigraphy and palaeomagnetism. The arrows at 3.27 Ma and 3.84 Ma are added for reference. This shows the level of sedimentary complexity highlighted between two inferred hiatus lines, with the projected ages of sediment above (3.27 Ma) and below (3.84 Ma) this zone highlighted with red arrows. The site shows a sharp change in sedimentation rate associated with a lithological change from calcareous to clastic dominated sedimentation. The transition interval is characterised by glauconite rich sediments that may represent the breaking-up of one or more hardgrounds (discussed further *ref. 20*).

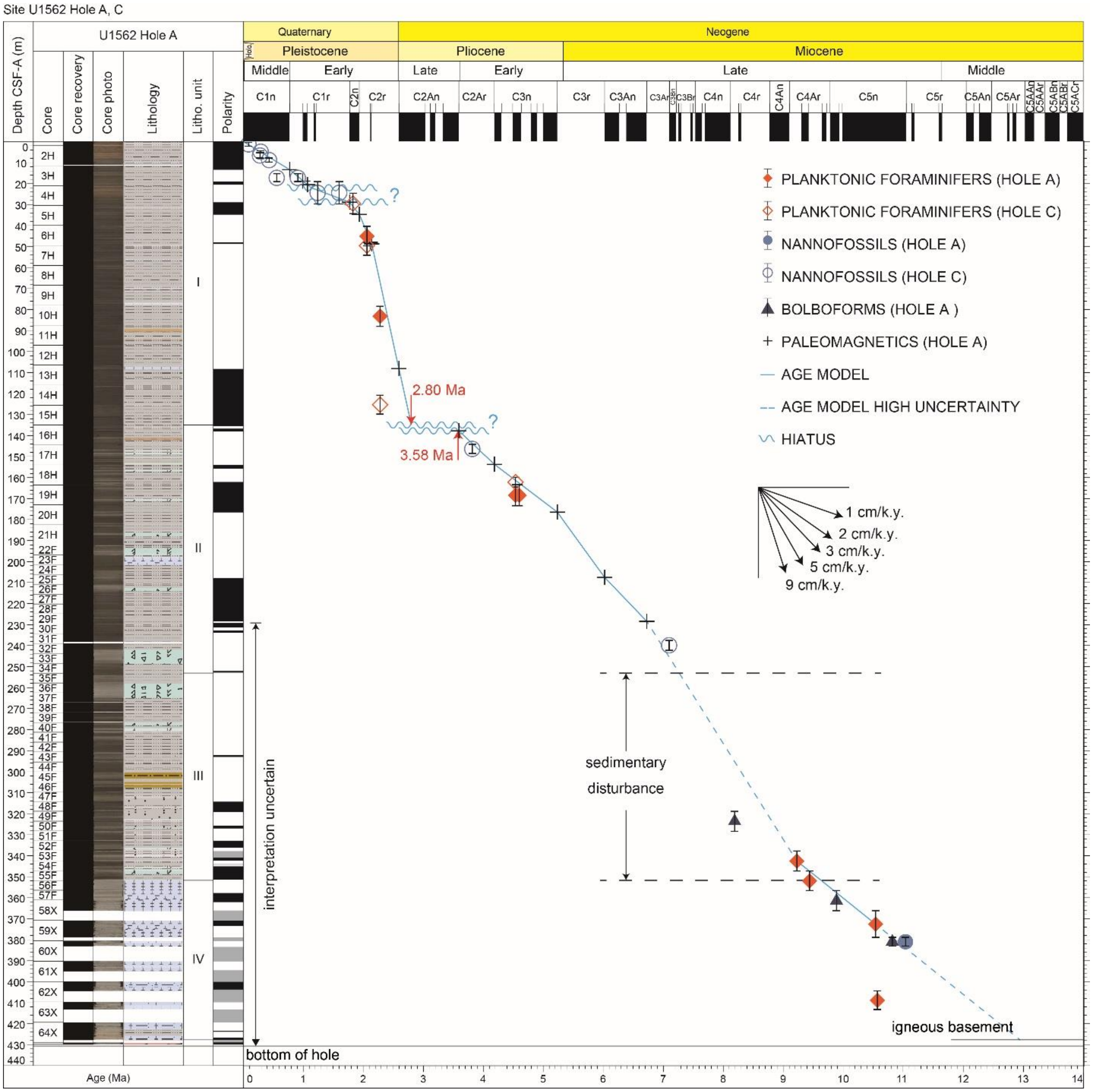


**Figure S5**: Age model for IODP Site U1562 based on biostratigraphy and palaeomagnetism, modified from *(21)*. The arrows at 2.80 Ma and 3.68 Ma are added for reference. This shows an inferred hiatus level that corresponds to a change in lithology, with the projected ages of sediment above (2.80 Ma) and below (3.58 Ma) this zone highlighted with red arrows. The site shows a sharp increase in sedimentation rate associated with a marked change in lithology. The transition is characterised by sediments rich in authigenic minerals (discussed further in *ref. 21*).

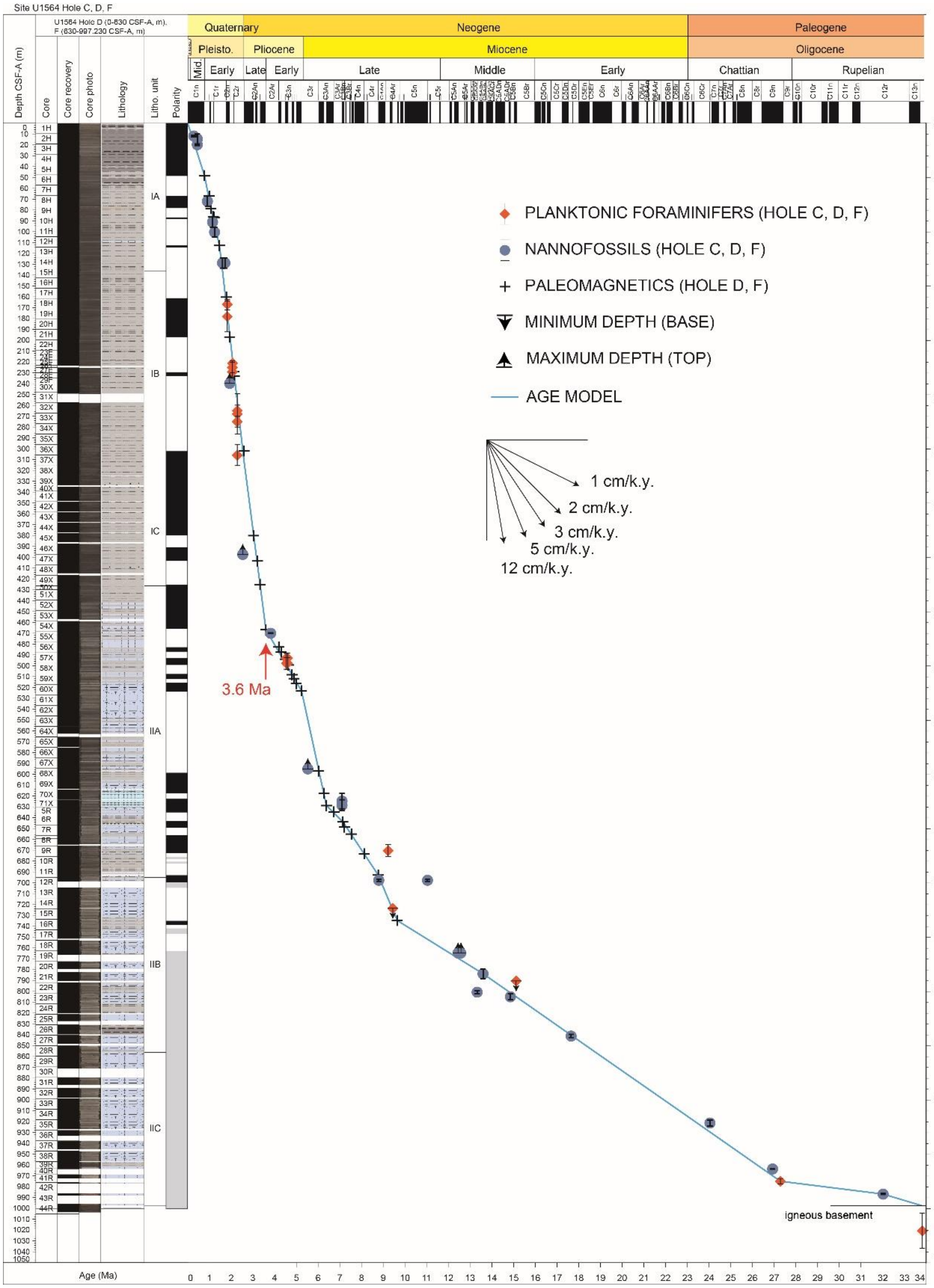


**Figure S6**: Shipboard age model for IODP Site U1564 based on biostratigraphy and palaeomagnetism. This shows an increase in sedimentation rate around 3.6 Ma highlighted with a red arrow (discussed further *ref. 22*). The precise timing of this change is explored further with an astronomically tuned age model as discussed in the following sections.

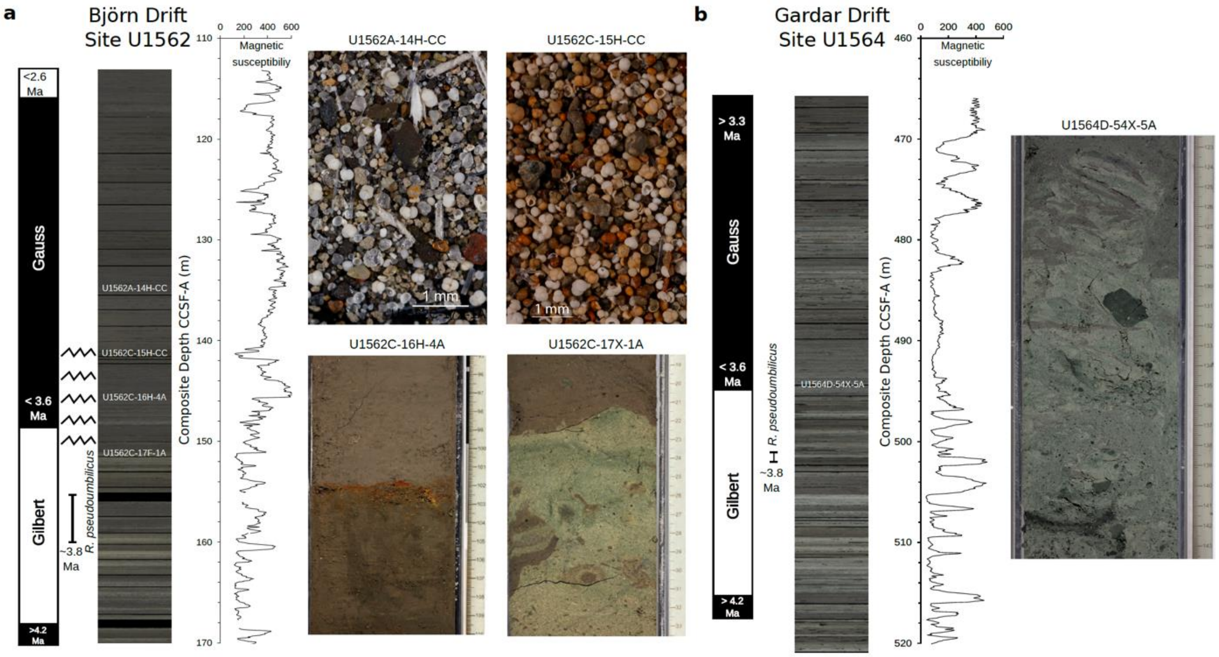


**Figure S7: Diagrams illustrating two styles of the sedimentation rate transition from a more slowly accumulating, carbonate rich facies into a faster accumulating, silty clay drift sediments. a,** The sedimentary record at Site U1562 is characterized by multiple sharp lithological boundaries indicating the presence of hiatuses (chevron pattern). **b,** At Site U1564, the transition only has minor indications of sedimentary disturbances. Core images of the composite record (splice) show the transition, with white labels indicating the position of adjacent close-up photographs. The magnetic susceptibility curve, shown on the core composite depth below seafloor (CCSF-A) scale, for Site U1564 illustrates the sedimentation rate transition where thinner light–dark cycles change into thicker cycles moving up the core. Image U1562A-14H-CC illustrates a post-transition foraminifer residue that is rich in ice-rafted debris (IRD). Image U1562C-15H-CC illustrates a glauconite-rich sample from the transition interval. Images U1562C-16H-4A and U1562C-17X-1A are illustrations of the sharp lithological transitions, suggesting hiatuses. Image U1564D-54X-5A illustrates sedimentary disturbance and presence of glauconite grains at the transition interval.

**Additional description of the sedimentary nature of the 3.6 Ma transition at Björn and Gardar Drifts**

All Björn Drift sites (U1554, U1562 and U1563) demonstrate a transition to faster drift sedimentation and a change from carbonate-rich facies to darker silty clay. The nature of the contact is, however, different to the Gardar site (U1564), in that Björn Drift Sites U1554 and U1562 contain more glauconite grains in the pre-transition sediments. At Site U1554 in the centre of the Björn Drift, there is a layer with abundant glauconitic grains immediately overlying the transition, indicating reworking of sediment. The same interval at Site U1562 on the drift edge shows a series of sharp lithological contacts suggesting condensation (**Fig. S5**), which is consistent with the pinching out of a significant part of the stratigraphic succession on the previously published seismic reflection profile (*14*). The timing of the transition at Site U1554 is very close to the base of the Gauss magnetochron and within error of the record at Gardar Drift Site U1564 (**Fig. S5**). The series of hiatuses at Site U1562 collectively represent several hundreds of thousands of years of missing sediment accumulation, making precise age determinations more difficult. A more continuous drift accumulation regime is only preserved starting around 2.8 Ma (**Fig. 2**).

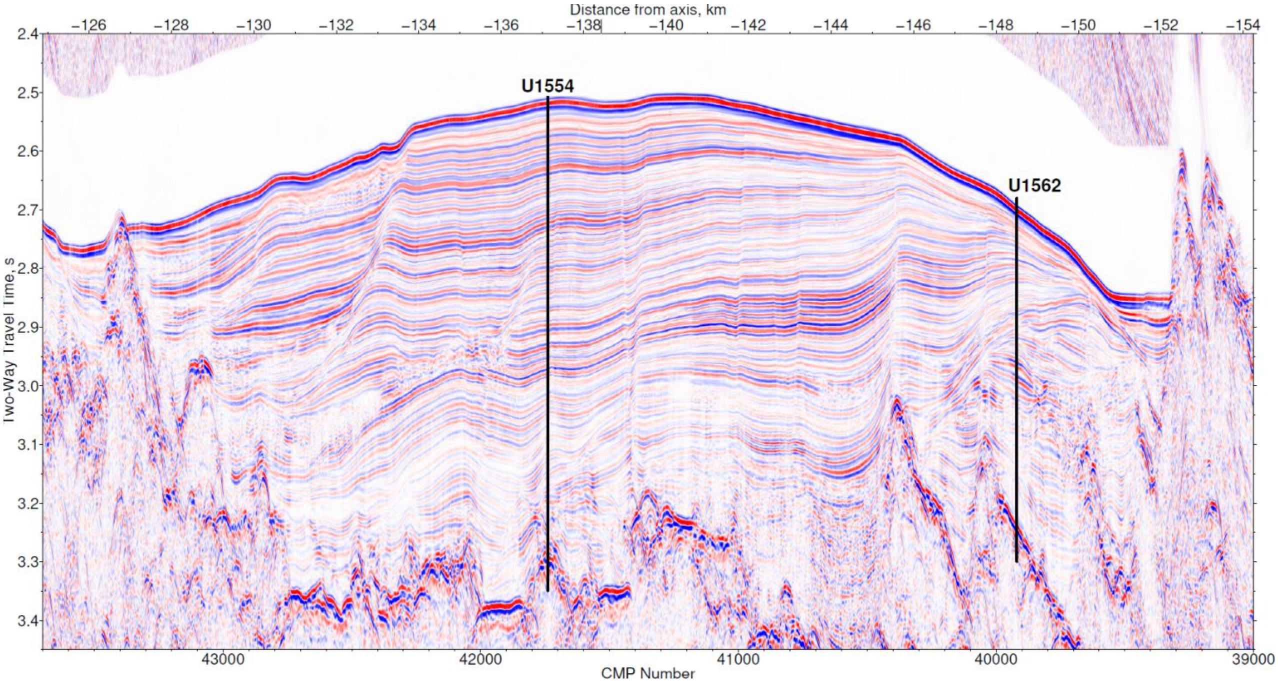


**Figure S8**: Seismic reflection profile for part of the Björn Drift with Site U1554 towards the centre and Site U1562 towards the eastern edge of the drift body, illustrating the pinching out of certain stratigraphic levels in Site U1562 relative to Site U1554 (*14*).

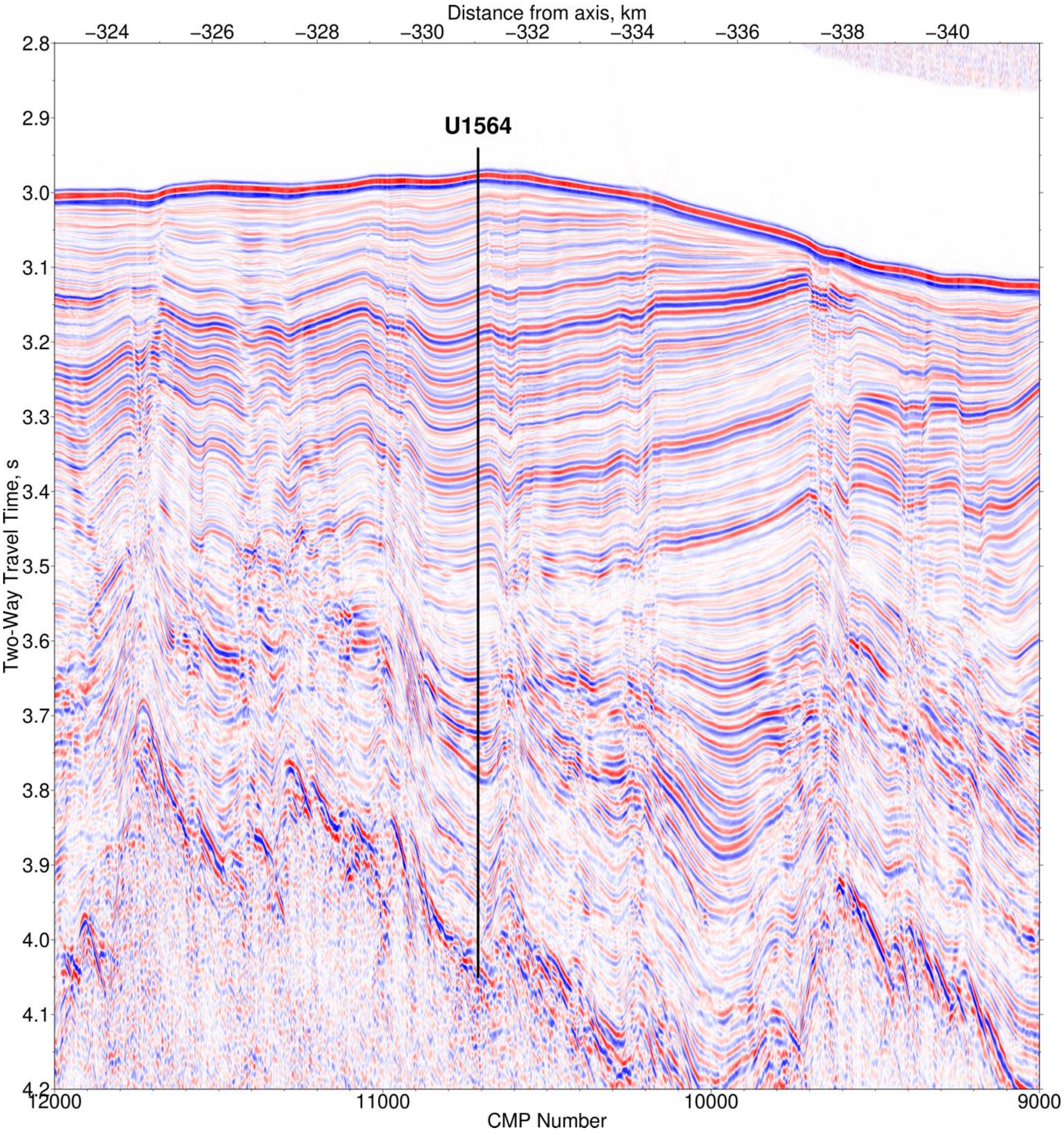


**Figure S9**: Seismic reflection profile for part of the Gardar Drift with Site U1564 illustrating the relatively undisturbed stratigraphic sedimentary succession (*14*).

**Construction of palaeomagnetic and astronomical time scale for U1564**

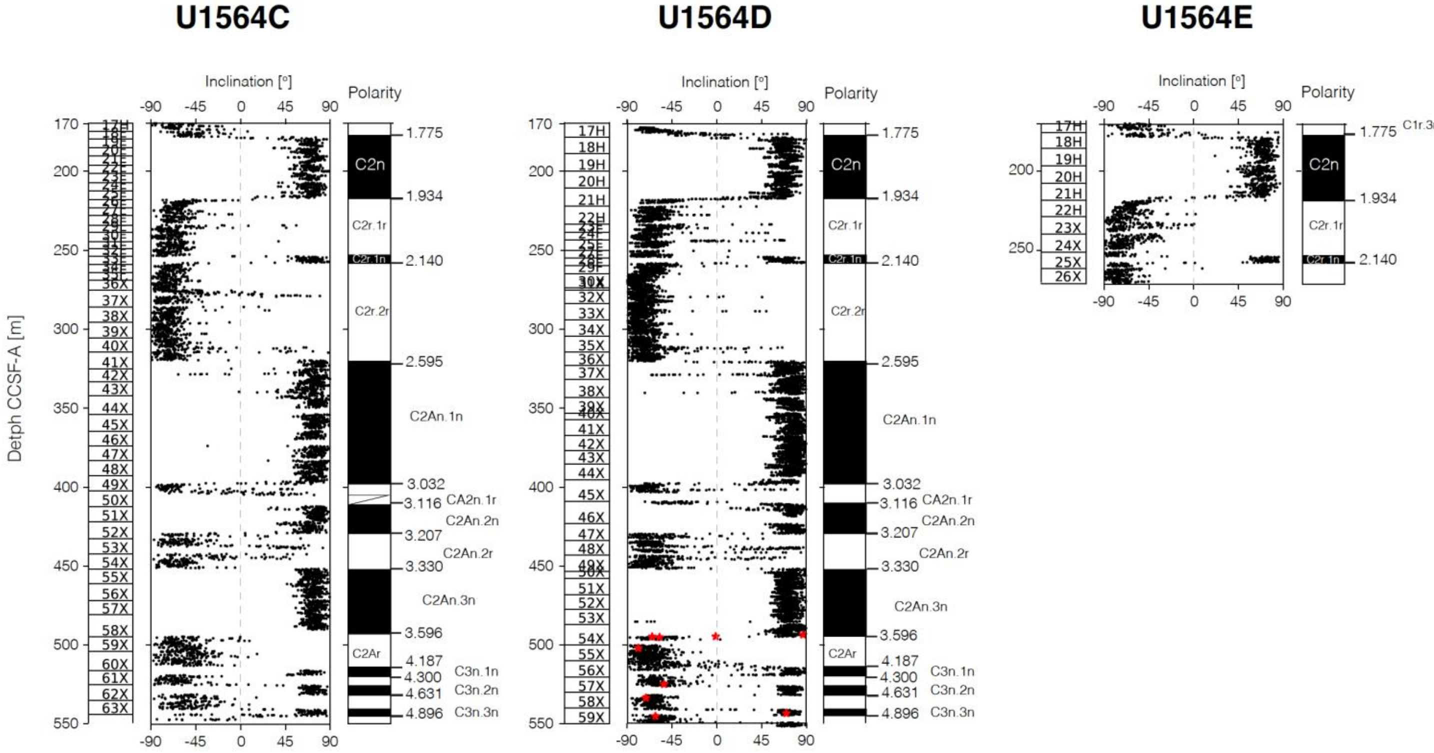


**Figure S10**: Palaeomagnetic inclination data (at 20 mT step) between 550 and 170 m CCSF-A (5-2 Ma) for Holes U1564C, U1564D and U1564E (*22*) that were used in the construction of the splice. Red stars for U1564D indicate shipboard measurements from discrete samples which confirm the robust identification of the 3.596 Ma transition from the C2Ar reversal to the C2An.3n normal chron.

Ocean Drilling Program (ODP) Site 983 also drilled on the Gardar Drift has a very similar stratigraphy to Site U1564 where they overlap. ODP Site 983 is well-studied in terms of magnetostratigraphy as well as Pleistocene glacial-interglacial variability (*12*, *15*). The deepest interval of ODP Hole 983C recorded the base of the Olduvai normal magnetochron. The excellent agreement in the magnetic susceptibility profiles between ODP Hole 983C (in meters composite depth) and the magnetitic susceptibility splice of Site U1564 (in m CCSF-A) is demonstrated in **Fig. S11**. This correlation is also consistent with the available magnetostratigraphic constraints that allow anchoring into the time domain. This temporal anchoring allows for the establishment of the phase relationship with low MS values corresponding with interglacial periods (or relative minima in the *ref. 16* benthic foraminifera oxygen isotope ratio stack (LR04)) and maxima in obliquity (**Fig. S11**). Age-depth tie points in addition to the magnetostratigraphic tie points for Site U1564 between 170 and 221 m CCSF-A were derived from the correlation between the relative minima in the LR04 stack and relative maxima in MS (**Fig. S11, Table `U1564_astronomical_tuning` in SI data**).

Below the interval cored by ODP Site 983, similarities in relative variations between the LR04 stack and MS are used for tuning, guided by the available palaeomagnetic reversal stratigraphy (**Fig. S12**). In some intervals these similarities are more pronounced (e.g. MIS 95 to 104) than in others (e.g. interval between 230-250 m CCSF-A without much variation in the MS profile). Below ~345 m CCSF-A the resemblance between the LR04 and MS profiles ends (MIS G7), and from there on the pronounced MS variations are tuned to the obliquity solution (as constrained by the position of the Gauss magnetozone) assuming the same phase relationship (**Figs. S12 and S13**). For the interval between 3.0 and 3.6 Ma, an additional precession signal seems to be expressed during relative obliquity minima and precession amplitude maxima (**Fig. S13**). The apparent missing obliquity cycle at 405 m CCSF-A in the core splice (not splice *strictu sensu*, there was a gap in the recovered material but the size of the gap was estimated using correlation with the downhole logging MS profile) MS record can actually be clearly identified in the downhole MS logging record. Tuning between 3.5 and 3.6 Ma is somewhat uncertain due to the sedimentary transition. However, there are no sedimentological indications that the sedimentation rate would be drastically different for magnetochron C2An.3n between 452 and 495 m CCSF-A. The sedimentary transition must be slightly younger than 3.596 Ma as constrained by the paleomagnetic reversal, but is most likely older than 3.55 Ma (allowing for the possibility of mistuning one obliquity cycle, or a minor erosional contact).

The tuning between 3.6 and 5.0 Ma assumed that every major wiggle in MS corresponds with an obliquity cycle, within palaeomagnetic and biostratigraphic age constraints (**Fig. S14**). Such first-order interpretation would mean a varied response of the climate and/or sedimentary system to the astronomical forcing with some cycles being much thicker and higher in amplitude than others. A possible better match could be obtained also considering variations in precession and resulting insolation, but no obvious better fits were obtained after trying various tuning targets. The presence of seven palaeomagnetic reversals between 514 and 550 m CCSF-A (or roughly 4.15-5.05 Ma) provides consistent and independent temporal constraints of the obliquity interpretation. The Top of *Reticulofenestra pseudoumbilicus* calcareous nannofossil horizon dated at 3.82 Ma (*17*) serves as useful extra stratigraphic control point between the 3.596 Ma and 4.187 Ma palaeomagnetic reversals, and its position was tightly constrained (501.23 ± 0.28 m CCSF-A) during the expedition by examining the biostratigraphy from additional in-core sampling.

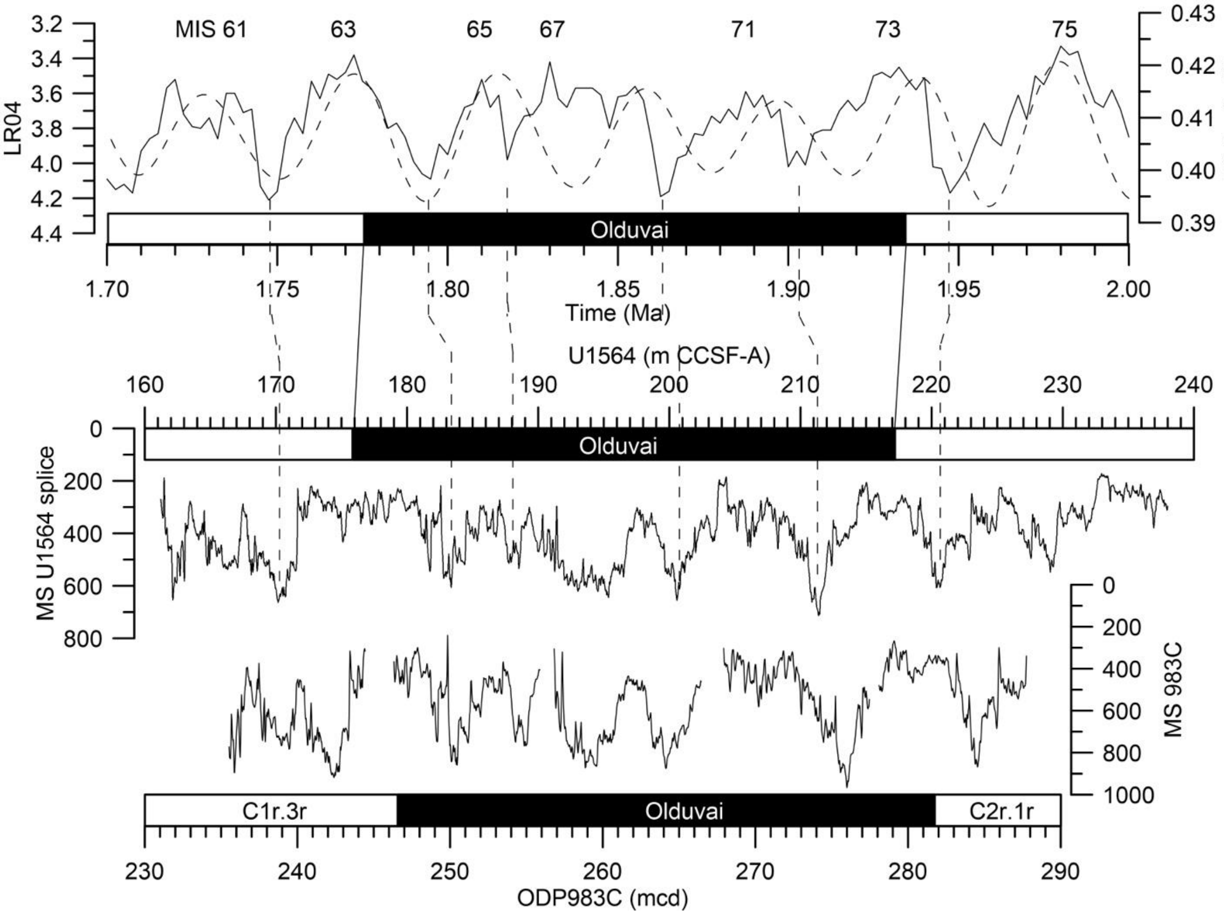


**Figure S11**: Correlation of similar MS profiles of overlapping stratigraphy of ODP Hole 983C with the Site U1564 splice further supported by palaeomagnetic data. Tuning with the LR04 stack using a phase relationship between high values in MS corresponding with the most positive benthic foraminifera oxygen isotopes (glacial periods) and relative minima in obliquity (dashed curve).

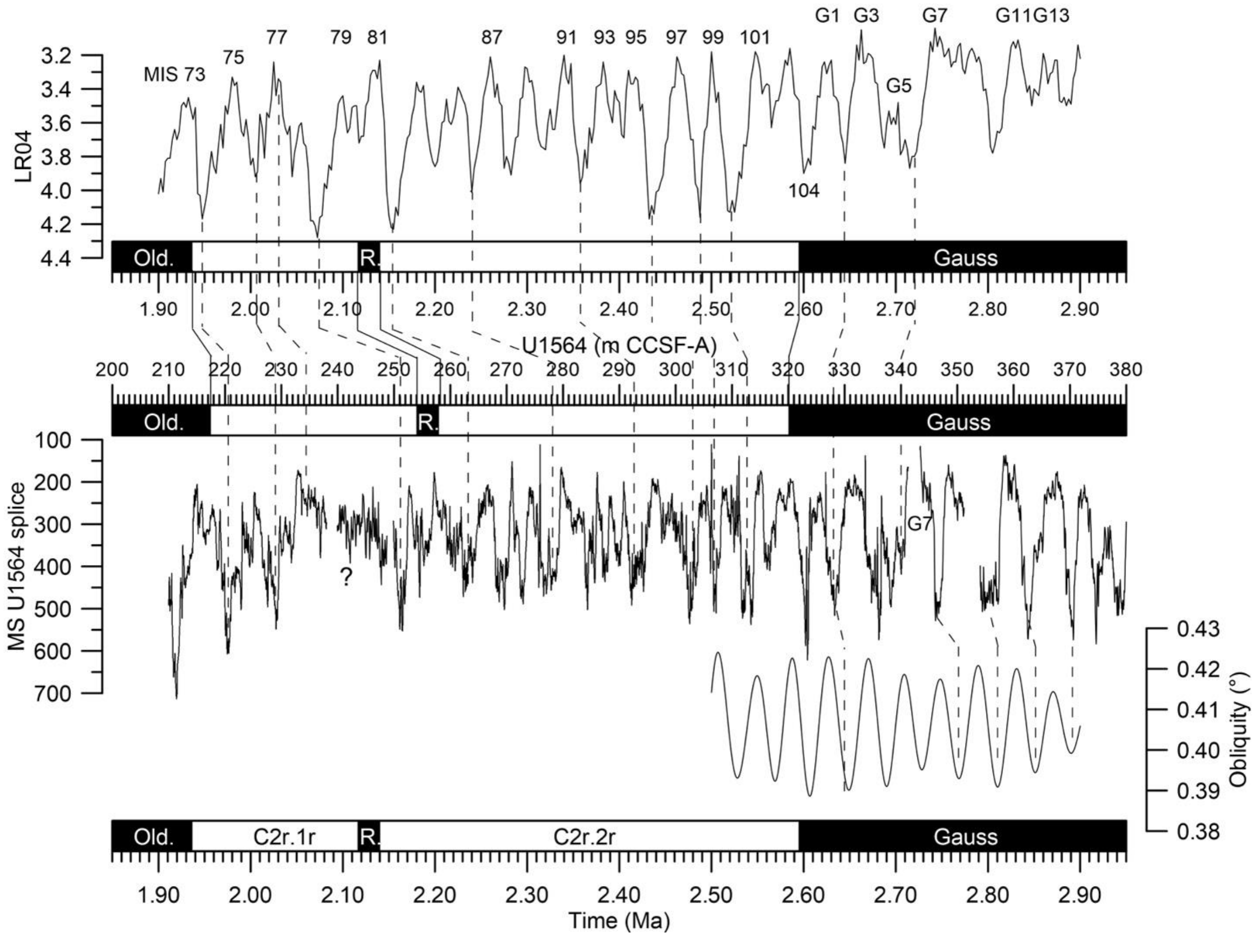


**Figure S12**: Astronomical tuning by correlating relative variations in MS and the LR04 stack between 1.9 and 2.7 Ma. The variations in MS in the stratigraphic interval older than 2.7 Ma shows little resemblance with the LR04 stack and thus are tuned to the obliquity solution (*23*), further constrained by the palaeomagnetic data.

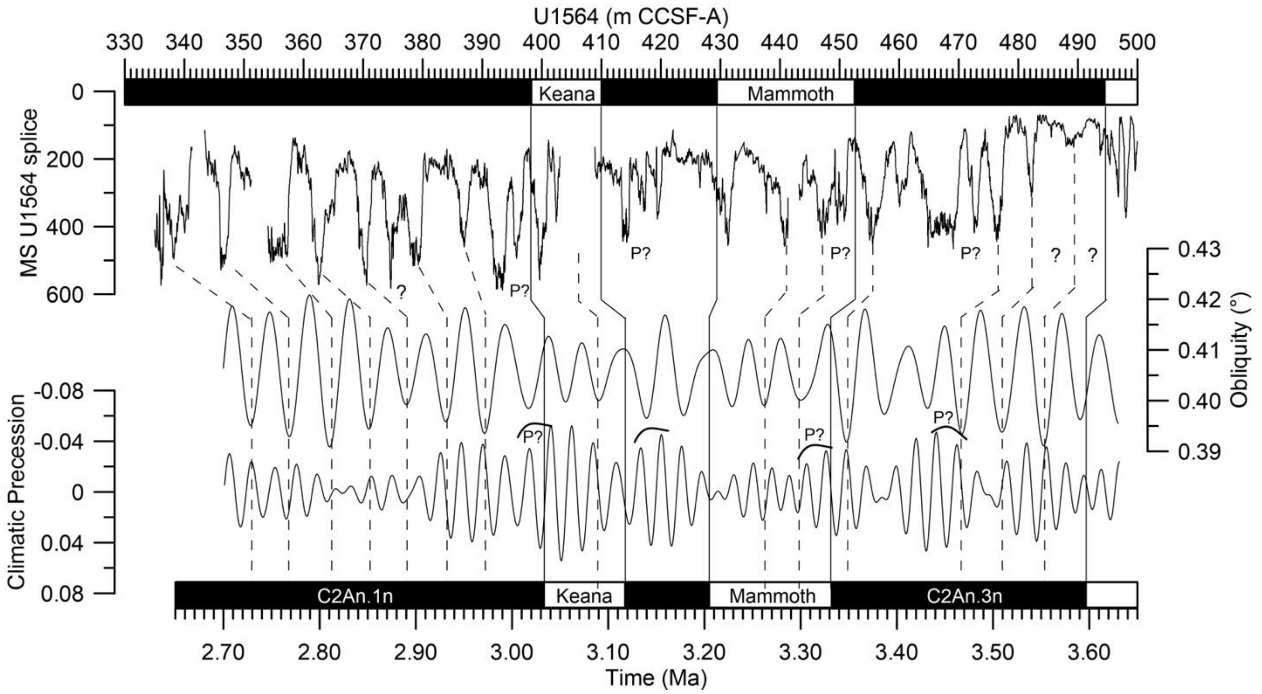


**Figure S13**: Astronomical tuning by correlating relative variations in MS to the astronomical solutions of obliquity and climatic precession (*23*), further constrained by the available palaeomagnetic data.

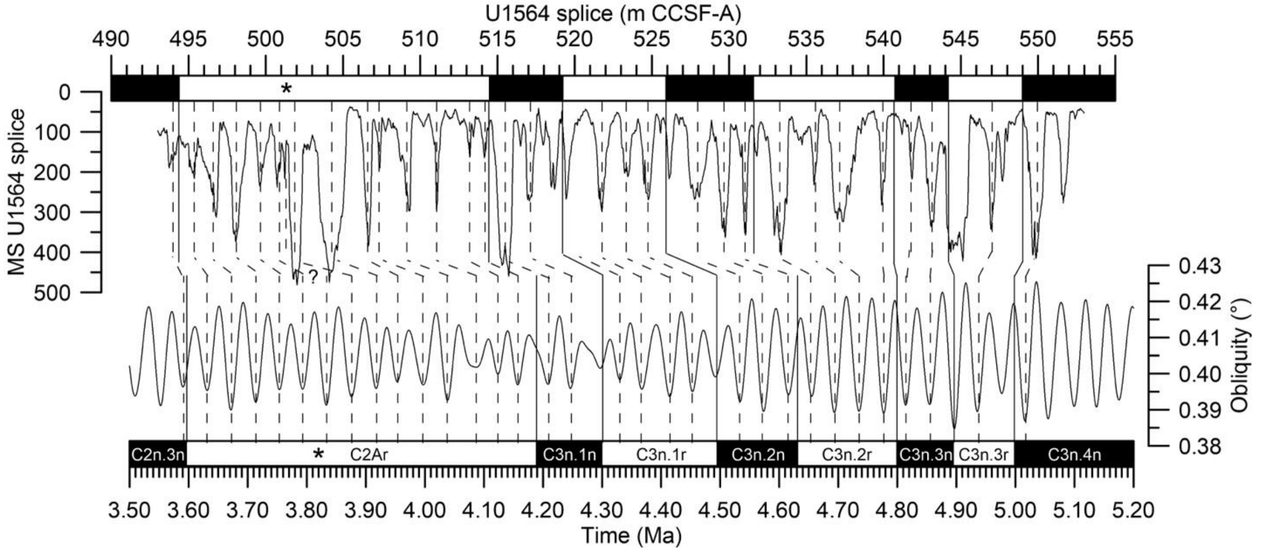


**Figure S14**: Astronomical tuning by correlating relative variations in MS to the astronomical solution of obliquity, further constrained by the available palaeomagnetic data. Top *R. pseudoumbilicus* nannofossil horizon is indicated by ‘*’.

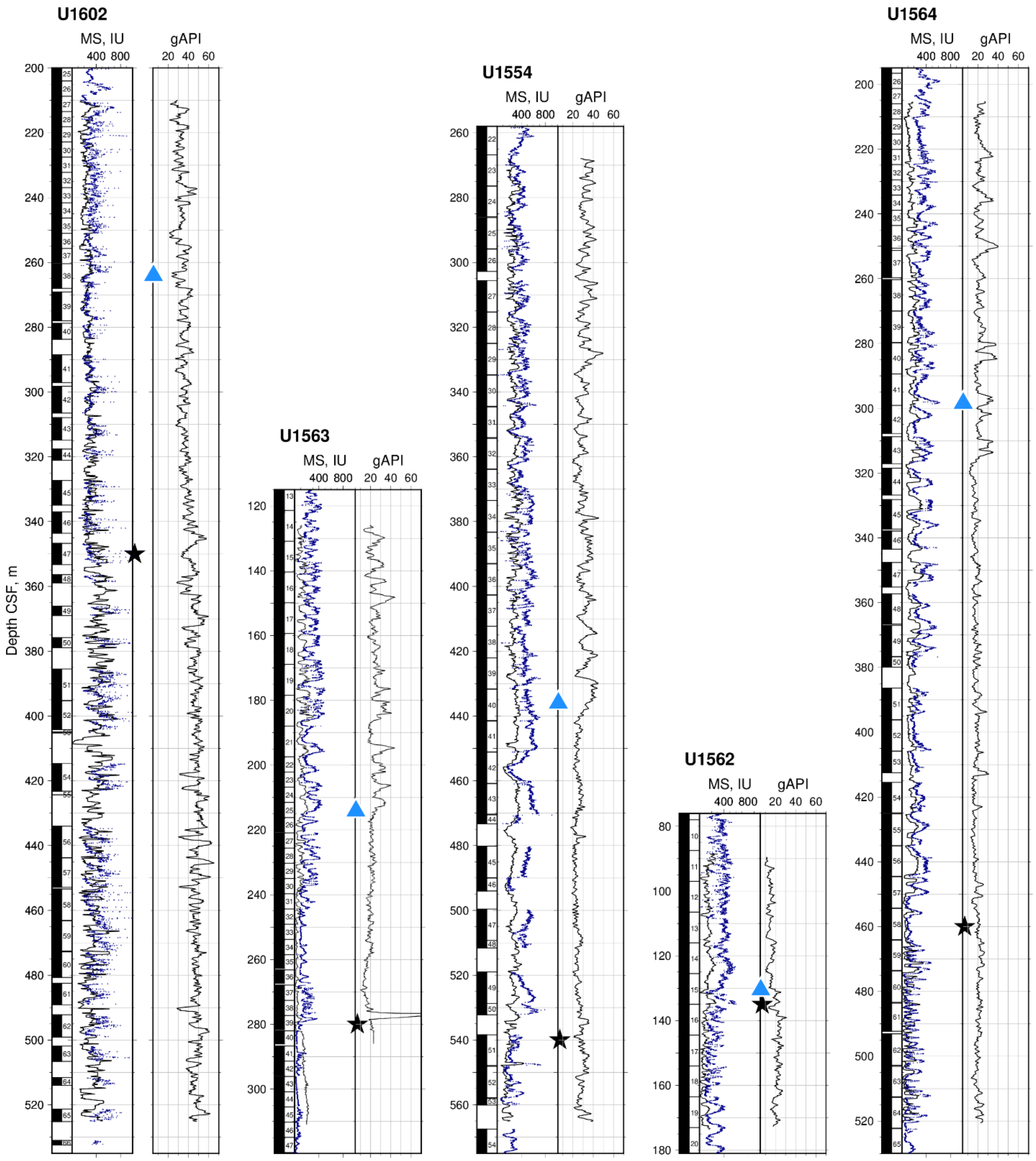


**Figure S15: Overview of stratigraphic geophysical changes in the studied sites.** Changes in magnetic susceptibility (MS; instruments units, IU) are measured both by half core point measurements (blue dots) and downhole logging (black lines). Natural gamma ray (gAPI) was measured by downhole logging (black lines). Black stars indicate the 3.6 Ma transition, with clear increases in magnetic susceptibility moving up section for Holes U1563A, U1554E and U1564D. Blue triangles correspond with the start of systematic occurrences of ice-rafted debris from foraminifer residues sampled from core catchers. Note that the stratigraphic position of the blue triangles is close to clear increases moving up section in natural gamma-ray for Holes U1563A, U1554E and U1564D. Black and white bars with numbers indicate core recovery and core numbers, respectively.

## SUPPLEMENTARY REFERENCES


1. Luyendyk B.P. *et al.* in *Proc. Init. Repts.*, DSDP 49, 21-160 (1979).

2. Jones, E.J.W., Ewing, M., Ewing, J.I. & Eittriem, S.L. Influences of Norwegian Sea overflow water on sedimentation in the northern North Atlantic and Labrador Sea. *J. Geophys. Res.* **75**, 1655-1680 (1970).

3. Ellett, D.J. & Roberts, D.G. The overflow of Norwegian Sea Deep Water across Wyville-Thompson Ridge. *Deep-Sea Res.* **20**, 819-835 (1973).

4. Ruddiman, W. F., Kidd, R. B., Thomas, E. *et al.* in *Proc. Init. Repts.*, DSDP 94, 351-590 (1987).

5. Kidd, R.B., & Hill, P.R. Sedimentation on Feni and Gardar sediment drifts. *In* Ruddiman, W.F., Kidd, R.B., *et al.*, *Init. Repts*. DSDP, 94 (Pt. 2), 1217-1244 (1987).

6. Srivastava, S. P., Arthur, M., Clement, B. *et al.* in *Proc. Init. Repts.*, ODP 105, 419-674 (1987).

7. Aubry, A.M.R., de Vernal, A. & Knutz, P.C. Baffin Bay late Neogene palynostratigraphy at Ocean Drilling Program Site 645. *Can. J. Earth Sci.* **58**, 67–83 (2021).

8. Jarrard, R.D. & Arthur, M.A. Milankovitch Paleoceanographic cycles in geophysical logs from ODP Leg 105, Labrador Sea and Baffin Bay. *In* Srivastava, S. P., Arthur, M., Clement, B. *et al.* in *Proc. Init. Repts. Sc. Res.*, ODP 105, 757-772 (1989).

9. Myhre, A.M., Thiede, J., Firth, J.V. *et al*. in *Proc. Init. Repts,* ODP 151, 57-111 (1995).

10. Larsen, H.C., Saunders, A.D., Clift, P.D. *et al.* in *Proc*. *Init. Repts,* ODP 154, 177-256 (1994).

11. St. John, K.E.K. & Krissek, L.A. The late Miocene to Pleistocene ice-rafting history of southeast Greenland. *Boreas* **31**, 28-35 (2002).

12. Jansen, E., Raymo, M.E., Blum, P. *et al.* in *Proc. Init. Repts.*, ODP 162, 49-387 (1996).

13. Channell, J.E.T. *et al*. *Proc. IODP*, Vol. 303/306 (2006).

14. Parnell-Turner, R. *et al.* Architecture of North Atlantic contourite drifts modified by transient circulation of the Icelandic mantle plume. *Geochem. Geophys. Geosyst.* **16**, 3414–3435 (2015).

15. Barker, S., *et al.* Persistent influence of precession on northern ice sheet variability since the early Pleistocene. *Science*, **376**, 961-967 (2022).

16. Lisiecki, L.E., & Raymo, M.E. A Pliocene-Pleistocene stack of 57 globally distributed benthic δ18O records. *Paleoceanography*, **20**, PA1003 (2005).

17. Raffi, I., Wade, B.S. & Pälike, H. The Neogene Period. In Gradstein, F.M., Ogg, J.G., Schmitz, M.D. & Ogg, G.M. (Eds.). Geologic Time Scale 2020, 1141–1215 (2020).

18. Parnell-Turner, R.E., Briais, A., LeVay, L.J. & the Expedition 395 Scientists. Site U1602. In Parnell-Turner, R.E., Briais, A., LeVay, L.J. & the Expedition 395 Scientists. Reykjanes Mantle Convection and Climate. Proceedings of the International Ocean Discovery Program, 395: College Station, TX (International Ocean Discovery Program).

https://doi.org/10.14379/iodp.proc.395.108.2025, (2025a).

19. Briais, A., Parnell-Turner, R.E., LeVay, L.J. & the Expedition 395 Scientists. Site U1563. In Parnell-Turner, R.E., Briais, A., LeVay, L.J. & the Expedition 395 Scientists. Reykjanes Mantle Convection and Climate. Proceedings of the International Ocean Discovery Program, 395: College Station, TX (International Ocean Discovery Program). https://doi.org/10.14379/iodp.proc.395.106.2025, (2025a).

20. Parnell-Turner, R.E., Briais, A., LeVay, L.J. & the Expedition 395 Scientists. Site U1554. In Parnell-Turner, R.E., Briais, A., LeVay, L.J. & the Expedition 395 Scientists. Reykjanes Mantle Convection and Climate. Proceedings of the International Ocean Discovery Program, 395: College Station, TX (International Ocean Discovery Program). https://doi.org/10.14379/iodp.proc.395.103.2025, (2025b).

21. Briais, A., Parnell-Turner, R.E., LeVay, L.J. & the Expedition 395 Scientists. Site U1562. In Parnell-Turner, R.E., Briais, A., LeVay, L.J. & the Expedition 395 Scientists. Reykjanes Mantle Convection and Climate. Proceedings of the International Ocean Discovery Program, 395: College Station, TX (International Ocean Discovery Program). https://doi.org/10.14379/iodp.proc.395.105.2025, (2025b).

22. Parnell-Turner, R.E., Briais, A., LeVay, L.J. & the Expedition 395 Scientists. Site U1564. In Parnell-Turner, R.E., Briais, A., LeVay, L.J. & the Expedition 395 Scientists. Reykjanes Mantle Convection and Climate. Proceedings of the International Ocean Discovery Program, 395: College Station, TX (International Ocean Discovery Program). https://doi.org/10.14379/iodp.proc.395.107.2025, (2025c).

23. Laskar, J., Robutel, P., Joutel, F., Gastineau, M., Correia, A.C.M. & Levrard B. A long-term numerical solution for the insolation quantities of the Earth. *A&A*, **428**, 261-285 (2004).
