# Supplementary material for: Onset of strong Iceland-Scotland overflow water 3.6 million years ago
Source: Nat Commun. 2025 May 9;16:4323. doi: 10.1038/s41467-025-59265-5 (PMC12064707; doi:10.1038/s41467-025-59265-5)
Supplement: Supplementary file 2 — Description of Additional Supplementary Files [file 41467_2025_59265_MOESM2_ESM.pdf]

Description of Additional Supplementary files

Onset of strong Iceland-Scotland Overflow Water 3.6 million years ago

Matthias Sinnesael[1,2*‡], Boris Th. Karatsolis[3,4*], Paul N. Pearson[5], Anne Briais[6], Sidney R. Hemming[7,8], Leah J. LeVay[9], Tom Dunkley Jones[10], Ying Cui[11], Anita Di Chiara[12], Justin P. Dodd[13], Deepa Dwyer[14], Deborah E. Eason[15], Sarah A. Friedman[16], Emma Hanson[10], Katharina Hochmuth[17,18], Halima E. Ibrahim[19], Claire E. Jasper[7,8], Saran Lee-Takeda[20], Danielle E. LeBlanc[21], Melody R. Lindsay[22], David D. McNamara[23], Sevasti E. Modestou[24], Margaret Morris[25], Bramley J. Murton[26], Suzanne OConnell[27], Gabriel Pasquet[28], Sheng-Ping Qian[29], Yair Rosenthal[30], Sara Satolli[31], Takuma Suzuki[32], Thena Thulasi[33], Bridget S. Wade[5], Nicholas J. White[34], Tao Wu[35], Alexandra Y. Yang[36] & Ross E. Parnell-Turner[25]

* These authors contributed equally to this work
‡ Corresponding author (sinnesam@tcd.ie)

1. File Name: SUPPLEMENTARY DATA

Description: This file contains the following supplementary records:

1) Hole summaries for IODP Expeditions 395C and 395

2) Stratigraphic distribution of quartz and rock fragments in palaeontological sample residues, interpreted as IRD

3) Stratigraphic distribution of glauconite grains in palaeontological sample residues

4) Lithological summaries for sites U1554, U1562, U1563, U1564 and U1602

5) Age-model tables and plots for sites U1554, U1562, U1563, U1564 and U1602

6) U1564 5-2 Ma astronomical tuning

2. File Name: SUPPLEMENTARY INFORMATION

Description: This file contains supplementary information regarding previously drilled sites in the North Atlantic and the Nordic Seas area, and the age-depth plots of the investigated sites. Additionally, it contains additional sedimentological, seismic and geophysical details regarding the proposed 3.6 Ma transition, as well as the development of an astronomically tuned age model for Site U1564.
